# Expression profiling across wild and cultivated tomatoes supports the relevance of early miR482/2118 suppression for *Phytophthora* resistance

**DOI:** 10.1098/rspb.2017.2560

**Published:** 2018-02-28

**Authors:** Sophie de Vries, Andreas Kukuk, Janina K. von Dahlen, Anika Schnake, Thorsten Kloesges, Laura E. Rose

**Affiliations:** 1 Department of Biochemistry and Molecular Biology, Dalhousie University, Halifax, Canada NS B3H 4R2; 2 Institute of Population Genetics, Heinrich-Heine University Duesseldorf, Universitaetsstr. 1, 40225 Duesseldorf, Germany; 3 iGRAD-Plant Graduate School, Heinrich-Heine University Duesseldorf, Universitaetsstr. 1, 40225 Duesseldorf, Germany; 4 Ceplas, Cluster of Excellence in Plant Sciences, Heinrich-Heine University Duesseldorf, Universitaetsstr. 1, 40225 Duesseldorf, Germany

**Keywords:** *Solanum*, miRNA signalling, evolution, plant immunity

## Abstract

Plants possess a battery of specific pathogen resistance (*R-*)genes. Precise *R-*gene regulation is important in the presence and absence of a pathogen. Recently, a microRNA family, miR482/2118, was shown to regulate the expression of a major class of *R-*genes*,* nucleotide-binding site leucine-rich repeats (*NBS-LRRs*). Furthermore, RNA silencing suppressor proteins, secreted by pathogens, prevent the accumulation of miR482/2118, leading to an upregulation of *R-*genes. Despite this transcriptional release of *R*-genes, RNA silencing suppressors positively contribute to the virulence of some pathogens*.* To investigate this paradox, we analysed how the regulation of *NBS-LRRs* by miR482/2118 has been shaped by the coevolution between *Phytophthora infestans* and cultivated and wild tomatoes. We used degradome analyses and qRT-PCR to evaluate and quantify the co-expression of miR482/2118 and their *NBS-LRR* targets. Our data show that miR482/2118-mediated targeting contributes to the regulation of *NBS-LRRs* in *Solanum lycopersicum.* Based on miR482/2118 expression profiling in two additional tomato species—with different coevolutionary histories with *P. infestans*—we hypothesize that pathogen-mediated RNA silencing suppression is most effective in the interaction between *S. lycopersicum* and *P. infestans*. Furthermore, an upregulation of miR482/2118 early in the infection may increase susceptibility to *P. infestans*.

## 1. Introduction

Resistance proteins (R*-*proteins) are fundamentally important in plant pathogen interactions. They recognize pathogen molecules, called effectors, which are secreted by pathogens to hijack plant immune responses [1,2]. Upon recognition, R-proteins trigger a pathogen-specific immune response [3,4]. Such immune responses include the hypersensitive response (HR), resulting in the release of reactive oxygen species, and can ultimately lead to cell death.

Misregulation of *R-*genes carries high fitness costs. Over-expression of *R-*genes in the absence of a pathogen can severely decrease fitness [5,6]. By contrast, insufficient *R-*gene expression during pathogen attack can allow for pathogen infection [7]. While several regulatory mechanisms are at play for different *R-*genes and R-proteins [8,9], negative regulation via small RNAs was proposed to globally buffer *R-*gene expression to avoid misregulation [10].

One example of negative regulation of *R-*genes, specifically of nucleotide-binding site leucine-rich repeats (*NBS-LRRs*), is suppression by the microRNA (miRNA) family miR482/2118 [11–13]. Targeting of miR482/2118 leads either to the degradation of *NBS-LRR* mRNA or to an inhibition of the translation of the corresponding mRNAs [14]. The family is one of the most labile miRNA families, displaying low sequence conservation, even between closely related species, despite its widespread presence in the plant kingdom [11,13,15]. Its diversity is in part a consequence of the amino acid variability of its target sequence [16].

A major pathogen of cultivated tomato (*Solanum lycopersicum*) is *Phytophthora infestans*. However, *P. infestans* not only infects crops but also their wild relatives [17–22]. Populations of wild tomato species, given that they are not subjected to breeding, have experienced different evolutionary histories with *P. infestans* compared to the cultivated tomato. In fact, wild tomatoes harbour *R*-genes that effectively contribute to the resistance to this pathogen [19,23].

*Phytophthora infestans*'s vast effector repertoire is likely the result of a constant adaptation to its diverse hosts [24]. Among *P. infestans*'s effectors, two were recently identified, which suppress the host's RNA silencing machinery [25–26]. Suppression of the plant RNA silencing pathways would release miRNA targets, including *NBS-LRRs*, from their miRNA-mediated suppression. Therefore, it has been hypothesized that the regulation of *R-*genes by miR482/2118 may have evolved into a pathogen detection mechanism, i.e. a counter-defence mechanism by which pathogen-mediated RNA silencing suppression activates the plant immune system [13]. This is at odds with the observed positive influence on pathogen virulence by these effectors [25] and suggests a complex network of *NBS-LRR* regulation during the infection of plants by their pathogens.

In this study, we analysed how coevolution of tomatoes and their pathogen *P. infestans* has shaped miRNA-mediated *NBS-LRR* regulation and how this regulatory network contributes to resistance in tomato. We first identified miR482/2118 targets associated with *P. infestans* defence in *S. lycopersicum.* Next, we studied the expression of *Sl*miR482/2118 and a set of 12 *NBS-LRRs* in *S. lycopersicum* during infection by *P. infestans*. Although the expression of *NBS-LRRs* is undoubtedly regulated by multiple mechanisms in addition to negative regulation via miRNAs, we observe examples of strong co-regulation between members of miR482/2118 and their targets. Combining comparative expression analyses of members of the miR482/2118 family in three closely related tomato species (*S. lycopersicum, Solanum pimpinellifolium* and *Solanum arcanum*) and analyses of host resistance led to two observations: (i) the least resistant tomato, *S. lycopersicum,* showed downregulation of several miRNAs from 24 to 96 hours post-inoculation (hpi) relative to the mock control, while its more resistant wild relatives did not and (ii) downregulation of miR482a and miR482f during early time-points of infection (6 hpi) correlated with resistance to *P. infestans.* Based on these observations, we hypothesize that global pathogen-mediated RNA silencing suppression is more effective in cultivated tomato than in its wild relatives.

## 2. Material and methods

### (a) Plant material and *Phytophthora infestans* inoculation

Seeds of *S. arcanum* were surface sterilized using approximately 5% NaOCl (30 s), washed 3×3 min in sterile H_2_O, plated on 1.2% H_2_O agar and incubated in dark for 3 days (16 h/8 h with 18°C/15°C). Afterwards, the seeds were transferred to a 16 L (166 ± 17 µmol quanta m^−2^ s^−1^) : 8 D regime. Nine days post sterilization (dps), seedlings were transferred to 0.5% Murashige & Skoog medium [27] with 1% sucrose.

The isolate, IPO-C, of *P. infestans* was grown on rye-sucrose-agar plates (with 100 µg ml^−1^ ampicillin, 10 µg ml^−1^ amphotericin B and 20 µg ml^−1^ vancomycin; [28]) at 18°C in the dark. Zoospores were isolated and leaflets of *S. arcanum* were inoculated at 28 dps as described in de Vries *et al*. [29]. Three biological replicates (three to four seedlings each) were sampled per treatment and time-point (0 hpi, 6 hpi, 24 hpi, 48 hpi, 72 hpi and 96 hpi).

### (b) RNA extraction, mRNA purification and cDNA synthesis

Total RNA of *S. arcanum* was isolated using the Universal RNA/miRNA Purification Kit (Roboklon, Berlin, Germany). RNA from *S. lycopersicum* and *S. pimpinellifolium* was used from de Vries *et al*. [29]. mRNA was purified using the Dynabeads mRNA Purification Kit (Thermo Scientific, Massachusetts, USA).

cDNA libraries for mature miR482/2118 expression analyses were created using miScript Plant RT Kit (Qiagen, Hilden, Germany) using 250 ng total RNA and diluted 1 : 10 with nuclease-free H_2_O. cDNA libraries for all other expression analyses were created with the RevertAid First Strand cDNA Synthesis Kit (Thermo Fisher Scientific, Vilnius, Lithuania) using 1000 ng total RNA and random hexamer primers and libraries were diluted 1 : 1 with nuclease-free H_2_O.

cDNA libraries for the modified 5'RNA ligase-mediated rapid amplification of cDNA ends (5′RLM-RACE) were created using the GeneRacer Kit (Invitrogen, California, USA) using 50–100 ng mRNA from infections (24 and 48 hpi) and mock (48 hpi). To identify miRNA cleavage sites, the protocol was modified to omit the enzymatic digest of the cap and proceed directly to the ligation of the 5′ GeneRacer RNA oligo adapter. The SuperScript III RT Module (Invitrogen, California, USA) with the GeneRacer Oligo dT Primer was used for reverse transcription.

### (c) 5′RLM-RACE

Amplification of 5′RLM-RACE products was performed (1× High Fidelity PCR buffer, 0.6 µM GeneRacer 5′ primer, 0.2 µM of the gene specific primer (electronic supplementary material, table S1), 200 µM dNTPs, 1 mM MgSO_4_, 3% DMSO and 0.5U Platinum *Taq* DNA Polymerase High Fidelity) followed by a nested PCR, using 1 µl of the PCR product in a 50 µl reaction (1× High Fidelity PCR buffer, 0.2 µM GeneRacer 5′ nested primer, 0.2 µM of the nested gene specific primer (electronic supplementary material, table S1), 200 µM dNTPs, 1 mM MgSO_4_ and 0.5U Platinum *Taq* DNA Polymerase High Fidelity).

PCR products were amplified with a Phusion High-Fidelity DNA Polymerase (New England Biolabs, Massachusetts, USA) and cloned using a Zero Blunt TOPO PCR Cloning Kit (Invitrogen, California, USA).

### (d) Confirmation of infection and infection progress

To confirm successful infection and study the disease progression, leaflets of *S. arcanum* seedlings were analysed microscopically. The relative necrotic area and pathogen structures were determined according to [29]. Statistical differences in necrotic area over time and between mock and infections were estimated using a Kruskal–Wallis test [30] with a Tukey and Kramer *post hoc* test, using a Tukey distance approximation [31]. For comparisons of the relative necrotic area between species, normal distribution of the data was evaluated using a Shapiro–Wilk test [32] and then tested for significant differences using a Mann–Whitney *U* test [33] in R v. 3.2.1. To determine the abundance and life cycle progression of *P. infestans* at the molecular level, expression of three biotrophic, two necrotrophic and one biomass marker gene were analysed according to [29].

### (e) Identification of miR482/2118 family members

Members of miR482/2118 from *S. lycopersicum* and *S. pimpinellifolium* have been previously identified in de Vries *et al*. [15]. Members of miR482/2118 from *S. arcanum* were identified via a BLASTn against the *S. arcanum* genome using miR482/2118 precursor sequences of *S. lycopersicum* as query. The best hits in *S. arcanum* were aligned to the *Sl*miR482/2118 precursor sequences and the mature miR482/2118 sequences were determined. Folding of *S. arcanum* miR482/2118 precursors into hairpins was predicted using RNAfold [34] (electronic supplementary material, figure S1).

### (f) Selection of *R-*genes

We chose *R-*genes that were (i) predicted to be targeted by one or more members of miR482/2118 and (ii) associated with resistance to *P. infestans*. The 52 potential miR482/2118 target genes [15] were used as queries for a BLASTn-search against the NCBI nr/nt database limited to *S. lycopersicum*. The best functional annotated BLAST hit (e.g. excluding hits to entire chromosomes) was recorded. Hits with an e-value of 0, query coverage greater than 90% and an identity greater than 85% to an *R*-gene associated with resistance against *P. infestans* in *S. lycopersicum* or the resistance gene analogues (RGA) complex were determined as likely to be associated with resistance to *P. infestans*.

### (g) qRT-PCR

qRT-PCR was performed using the miScript SYBR Green PCR (Qiagen, Hilden, Germany). miR482/2118 forward primers were designed based on the mature miR482/2118 sequences. miR482/2118 primer specificity was tested by creating a qRT-PCR product for each primer. These qRT-PCR products were purified, and each primer was tested with each qRT-PCR product to determine if and at what annealing temperatures the primers would bind to other miR482/2118 paralogues. For all miR482/2118 primers a binding-specific annealing temperature was determined (electronic supplementary material, table S1). The only exceptions were the primers for *Sl*miR482h and *Sp*miR482h, which annealed to miR482h as well as miR482 at all annealing temperatures. *Sa*miR482h was specific because of its slightly different mature miRNA sequence (electronic supplementary material, table S1). As a control, the expression of mature *Sl*miR156a/b/c, *Sl*miR166a/b, *Sl*miR168a/b and *Sl*miR172a/b was determined. miR390a was used as a reference due to its constant expression across treatments and time-points according to BestKeeper v.1 [35].

Expression of *NBS-LRRs* in *S. lycopersicum* was determined using the SsoAdvanced Universal SYBR Green Supermix (electronic supplementary material, table S1; Bio-Rad, California, USA). As reference genes, we used *SAND* [15], *TIP41* [15] and *Translation Initiation Factor 3 subunit H* (*TIF3H;* [29]).

Relative abundance and progression of *P. infestans* were measured using *Histone2a* (*PiH2a*). Expression of *PiH2a* at time-points 24 to 96 hpi was set relative to its expression at 24 hpi. The data were normalized with the plant reference genes (*SAND, TIP41* and *TIF3H*).

Relative expression was calculated according to [36]. Data were tested for normality using a Shapiro–Wilk test [32] and equal variance using R v. 3.2.1. Comparisons between infections and mock control were tested using either a two-sample *t*-test or a Welch two-sample *t*-test for normally distributed data or a Mann–Whitney *U*-test [33] for non-normally distributed data.

## 3. Results and discussion

### (a) One-third of potential nucleotide-binding site leucine-rich repeats targets have high identity to *Phytophthora infestans*-associated resistance genes

We screened for *NBS-LRR*s that are potential targets of miR482/2118 and classified as *R-*genes for *P. infestans* (electronic supplementary material, table S2). Of the 52 predicted *NBS-LRR* targets [15], we identified 20 which were annotated as a *P. infestans*-associated *R*-gene or the *RGA* complex, members of which are associated with resistance to the pathogen [37–38]. Of these 20, 17 matched a *P. infestans-*associated *R-*gene with an e-value of 0, a query coverage of greater than 90% and an identity of greater than 85% (electronic supplementary material, table S2). Therefore, approximately 33% of the predicted direct *NBS-LRR* targets of miR482/2118 are associated with resistance to *P. infestans*.

### (b) Nucleotide-binding site leucine-rich repeats are targeted by miR482/2118 in *Solanum lycopersicum* during infection by *Phytophthora infestans* infection

**Figure 1.**
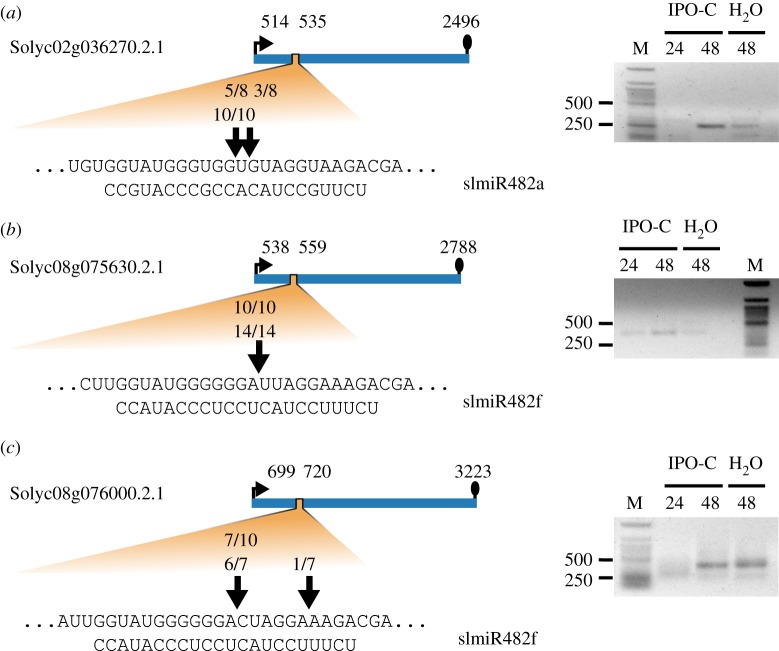
Targeting of *NBS-LRRs* by miR482/2118 family members in *S. lycopersicum. In vitro* confirmation of *NBS-LRR* targeting by *Sl*miR482/2118 using 5′RLM-RACE for *Solyc02g036270.2.1* targeted by *Sl*miR482a (*a*), *Solyc08g075630.2.1* targeted by *Sl*miR482f (*b*) and *Solyc08g076000.2.1* targeted by *Sl*miR482f (*c*). A schematic of the target gene (blue) is on the left. The predicted binding site (P-loop, orange) and its sequence is shown below. The arrows indicate the validated degradation sites. The number of clones supporting the site and the total number of clones sequenced are given above the arrows. Upper numbers indicate clones from the mock controls and lower numbers indicate those from infections. The corresponding PCR products of the 5′RLM-RACE are shown on the right.

Previous studies have used 5′RLM-RACE to test whether the expression of *NBS-LRRs* is regulated by members of the miR482/2118 gene family [13–14]. Targeting by *Sl*miR482f of *Solyc08g075630.2.1* and *Solyc08g076000.2.1,* which are associated with *P. infestans* defence responses (electronic supplementary material, table S2), was only shown in overexpression lines of *Nicotiana benthamiana* [14]. To test whether these *NBS-LRRs* are targeted by miR482/2118 in *S. lycopersicum,* we created 5′RLM-RACE libraries from *S. lycopersicum* infected with the pathogen and mock-treated (figure 1). In addition, we tested *Solyc02g036270.2.1*, because it is a functional miR482/2118 target [13] that is not associated with *P. infestans* resistance (figure 1; electronic supplementary material, table S2).

A cleavage site is determined by an enrichment of a specific degradation product in the 5′RLM-RACE library. This is established by cloning the degradation products of the gene of interest from the library and analysing how often a specific degradation product was cloned. If the gene of interest has a miRNA cleavage site, the majority of the clones should contain a product cut at the predicted cleavage site. All three tested genes revealed a cleavage site in the region complementary to the miR482/2118 sequences. Moreover, these cleavage products were observed in both mock-treated and *P. infestans* infected leaflets of *S. lycopersicum*. Based on clone analyses of the 5′RLM-RACE library of *Solyc02g036270.2.1,* 15 out of 18 clones were cleaved between nucleotide positions 11 and 12 of the miRNA binding site (figure 1*a*). For *Solyc08g075630.2.1*, all clones (24/24) were cleaved between nucleotide positions 12 and 13 of the miRNA binding site (figure 1*b*). For *Solyc08g076000.2.1*, 13/17 clones had a cleavage site between nucleotide positions 12 and 13 of the miRNA binding region (figure 1*c*). Some alternative cleavage products were observed for *Solyc02g036270.2.1* and *Solyc08g076000.2.1* (figure 1)*.* This is in agreement with Ouyang *et al*. [14], who also observed an alternative cleavage site for *Solyc08g076000.2.1*. In summary, we demonstrate that targeting of *NBS-LRRs* by miR482/2118 is effective in pathogen-challenged and unchallenged plants.

### (c) Co-regulation of members of miR482/2118 and their nucleotide-binding site leucine-rich repeats targets is time-dependent

**Figure 2.**
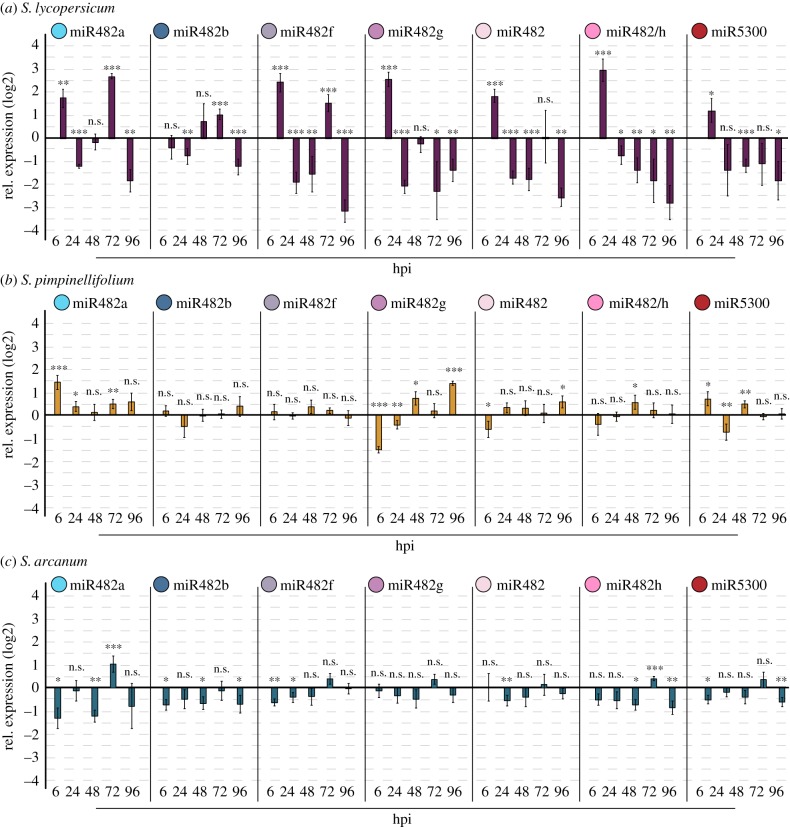
Expression of miR482/2118 family members in *S. lycopersicum, S. pimpinellifolium* and *S. arcanum.* Relative expression (log2) in infected compared with mock-control plants of *S. lycopersicum* (*a*), *S. pimpinellifolium* (*b*) and *S. arcanum* (*c*) of the seven miR482/2118 family members at 6, 24, 48, 72 and 96 hpi relative to mock control. The bars represent the average relative expression of the mature miRNAs and the error bars indicate the standard error of the mean (SEM). Significant differences of the relative expression of the miRNA in infected versus mock-treated plants at a specific time-point are indicated by *(*p*-value < 0.05), **(*p-*value < 0.01), ***(*p-*value < 0.001) and ns (not significant).

**Figure 3.**
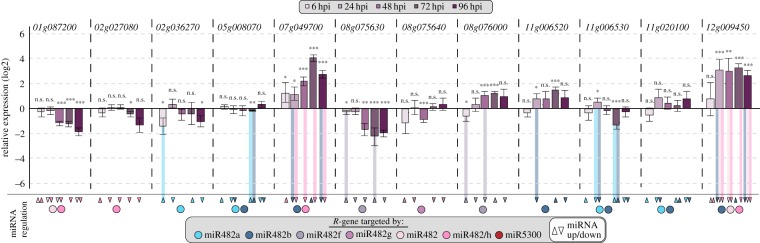
Expression and co-regulation of *Sl*miR482/2118 and their *NBS-LRR* targets. Relative expression (log2) of potential *NBS-LRR* targets of miR482/2118 in infected compared with mock-control plants of *S. lycopersicum*. Bars show the mean expression and error bars indicate the SEM. Statistical differences in relative expression in infected versus mock-treated plants at a specific time-point are indicated by *(*p-*value < 0.05), **(*p-*value < 0.01), ***(*p-*value < 0.001) and ns (not significant). Filled circles below each gene corresponds to the miRNA(s) predicted to target each *NBS-LRR.* Arrow heads indicate significant up or downregulation of the members of *Sl*miR482/2118 at a given time-point: upward arrow heads indicate significant upregulation and downward arrow heads indicate significant downregulation of the miRNA. The arrow heads are coloured according to their respective miRNA. Vertical lines between miRNA arrow heads and the relative expression of the *NBS-LRR* highlight significant negative co-regulation between members of the *Sl*miR482/2118 family and their targets at a specific time-point.

Given that a third of the miR482/2118 potential targets in *S. lycopersicum* are associated with disease resistance to *P. infestans* in *S. lycopersicum,* we chose a subset of 11 *NBS-LRRs* associated with *P. infestans* resistance and *Solyc02g036270.2.1* (as a positive control for cleavage, but a negative control in terms of *P. infestans* resistance) to study the co-regulation of *NBS-LRRs* and miR482/2118 in this interaction. We quantified the expression of the seven members of miR482/2118 and 12 *NBS-LRRs* in infected and uninfected plants across five time-points (6 to 96 hpi) (figures 2*a* and 3).

To identify to what degree the miRNAs show similar expression patterns in response to infection, we compared the expression of the individual miRNAs and recorded how often two miRNAs showed the same expression pattern in parallel at a given time-point, to see whether both show (i) significant upregulation, (ii) significant downregulation or (iii) no differential regulation between infection versus mock. Overall, all *Sl*miR482/2118 miRNAs show similar dynamics in expression, with the same expression pattern of two miRNAs for 3.1 ± 0.9 time-points, on average (figure 2*a*).

By contrast, two *NBS-LRRs* show, on average, the same expression pattern at 2.0 ± 1.3 time-points (figure 3). This is a significantly lower co-regulation compared to that observed for miR482/2118 (*p-*value = 0.0002). Such differences in co-regulation suggest that despite active targeting by miR482/2118 in *S. lycopersicum*, *NBS-LRRs* are likely to be regulated by other mechanisms in addition to the regulation by miR482/2118.

Next, we evaluated how often pairs of miR482/2118 and *NBS-LRRs* are co-regulated and what type of co-regulation they are subjected to (i.e. negative co-regulation, positive co-regulation or no differential regulation of both miRNA and target). In total (over all time-points), we evaluated 95 miR482/2118–*NBS-LRR* combinations (electronic supplementary material, figure S2). In 45 pairs, the *NBS-LRRs* are predicted to be post-transcriptionally regulated, while 50 are predicted to be translationally regulated (electronic supplementary material, table S2). If a target is post-transcriptionally regulated, one would predict a negative co-regulation of target and miRNA. This means that if the miRNA is significantly upregulated, the target should be significantly downregulated and vice versa. Nevertheless, positive correlations between miRNA and target mRNA levels have been reported [39–41]. Additionally, positive co-regulation has been observed for miRNAs [39] that suppress their targets translationally [42], suggesting that translational repression can lead to positive co-regulation. If the miRNA is not differentially regulated between infection versus mock treatment, the target should not be either.

We observed that the direction of co-regulation is not static for every miR482/2118–*NBS-LRR* combination but can shift between time-points. Such rapid shifts in co-regulation may result from switches between translational and post-transcriptional suppression. For example, *Solyc08g076000.2.1* shows an alternating pattern of co-regulation with *Sl*miR482f (figure 3; electronic supplementary material, S2) and is regulated by both modes [14], despite its prediction to be regulated translationally (electronic supplementary material, table S2).

We determined at which time-points co-regulation was most prevalent, suggesting a potential influence of miR482/2118 on *NBS-LRR*-regulation. The greatest co-regulation occurred at 48 hpi with 10/12 *NBS-LRRs* showing co-regulation with at least one of their respective *Sl*miR482/2118 members (electronic supplementary material, figure S2). High co-regulation was also detected at 6 and 72 hpi for 9/12 *NBS-LRRs*. All three time-points are biologically interesting: 6 hpi is a crucial time-point for infection success, as early HR significantly contributes to resistance against *P. infestans* [20]. Between 48 and 72 hpi, *P. infestans* switches from a biotrophic (i.e. requiring nutrients from a living host) to a necrotrophic phase (i.e. inducing host cell death) [29].

### (d) *Solanum arcanum* is less susceptible to *Phytophthora infestans* than its two relatives

We found that co-regulation of miR482/2118 with their targets was time-dependent, and more prevalent at time-points critical for infection success and transitions in the pathogen's life cycle*.* To place this in context with resistance, we compared the response of three tomato species, *S. lycopersicum, S. pimpinellifolium* and *S. arcanum,* to *P. infestans*. These host species differ in their evolutionary and ecological histories. *S. lycopersicum* has long been subjected to artificial selection. Furthermore, high-density monocultures of crop species can allow for higher pathogen loads and potentially higher pathogen diversity in the cultivated species [43].

*Solanum pimpinellifolium* and *S. arcanum* have partially overlapping ranges: *S. pimpinellifolium*'s habitat spans from Central Ecuador to Chile, while *S. arcanum* occurs in Northern Peru [44]. Furthermore, their habitats overlap with that of *P. infestans* [45–47], allowing for exposure to and coevolution with the pathogen. Indeed, *R*-genes associated with resistance to *P. infestans* have been isolated from *S. pimpinellifolium* [23,48]. In addition, *S. pimpinellifolium* is facultative self-compatible and *S. arcanum* is predominantly self-incompatible [44]. Mating system differences can influence the evolutionary history of the hosts and their adaptation potential*.* We therefore hypothesize that the different hosts will show variation in their resistance to *P. infestans* because they experienced different evolutionary histories.

**Figure 4.**
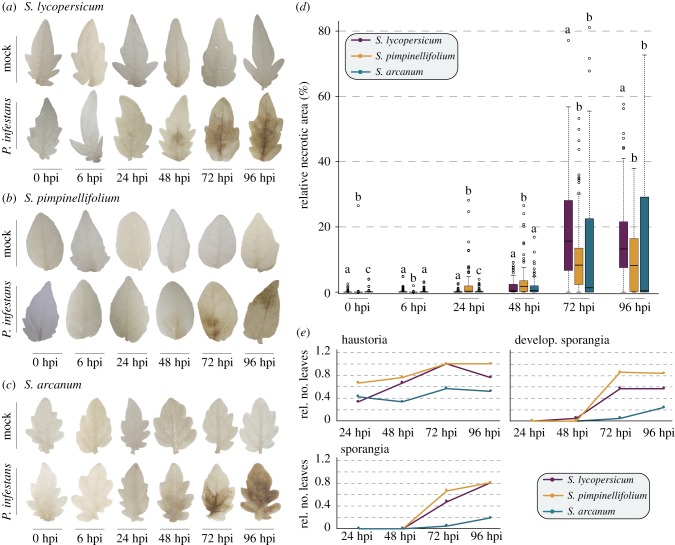
Infection progress in *S. arcanum* in comparison to *S. lycopersicum* and *S. pimpinellifolium.* Necrotic area on the leaflets of *S. lycopersicum* (*a*), *S. pimpinellifolium* (*b*) and *S. arcanum* (*c*) for mock-treated (upper row) and infected (lower row) leaflets. Comparison of the relative necrotic area during *P. infestans* infection in *S. arcanum* (blue)*, S. pimpinellifolium* (yellow) and *S. lycopersicum* (purple) (*d*)*.* Statistical differences in relative necrotic area for the three species were calculated per time-point and are indicated by different letters above the boxes. The *p-*value cut-off was 0.05. Comparison of the number of haustoria, developing and mature sporangia of *P. infestans* after infection of *S. arcanum* (blue), *S. pimpinellifolium* (yellow) and *S. lycopersicum* (purple) (*e*)*.* All data for *S. pimpinellifolium* and *S. lycopersicum* were published previously in de Vries *et al*. [29].

In our previous study [29], we evaluated the relationship between pathogen abundance, the presence of pathogen infection structures and disease symptoms in *S. lycopersicum* and *S. pimpinellifolium*. Here, we describe our new results on *S. arcanum* and compare these with the results from *S. lycopersicum* and *S. pimpinellifolium*. The relative necrotic area of *S. arcanum* increased significantly at 48 hpi (figure 4*c*; electronic supplementary material, S3a). Although the variance of relative necrotic area was higher in 72 and 96 hpi compared with 48 hpi, the relative necrotic area did not increase significantly beyond 48 hpi (electronic supplementary material, figure S3a). The abundance of *P. infestans* increased significantly from 24 to 48 hpi, and from 48 to 72 hpi (electronic supplementary material, figure S3b). The lack of correlation between relative necrotic area and *P. infestans* abundance at 72 hpi may stem from a delayed transition to the necrotrophic phase. For *S. pimpinellifolium* and *S. lycopersicum* we pinpointed the transition from biotrophy to necrotrophy to a time between 48 and 72 hpi [29]. For *S. arcanum,* we observed haustoria from 24 hpi onwards, and developing and mature sporangia at 72 and 96 hpi (electronic supplementary material, figure S3c). In agreement with this, most marker genes for biotrophy are expressed throughout the infection, but the sporulation marker *Cdc14* was only expressed from 72 hpi onwards (electronic supplementary material, figure S3d,e), suggesting that the transition to necrotrophy occurred between 48, 72 hpi. However, the number of all infection structures was lower in *S. arcanum* compared with the other two species (figure 4*e*). As less virulent isolates of *P. infestans* also show a reduction in haustoria compared with more virulent isolates [49], this suggests that *P. infestans* is less infective and has a delayed life cycle transition on *S. arcanum*.

Across all species, sporangia develop the earliest (48 hpi) in *S. lycopersicum* (figure 4*e*). The relative necrotic area 72 and 96 hpi is also the highest in *S. lycopersicum* (figure 4*a–d*). Taken together, this suggests that, although all species are susceptible to *P. infestans,* they are so by a variable degree: *S. lycopersicum* is likely the most susceptible, followed by *S. pimpinellifolium* and finally *S. arcanum,* which is the least susceptible of all three species.

### (e) MiR482a and miR482f are candidate miRNAs for defence responses against *Phytophthora infestans*

We evaluated the miRNA expression between the tomatoes in relation to their resistance phenotype. Compared with *S. lycopersicum,* expression between pairs of miRNAs was significantly less correlated in the wild tomatoes: in *S. pimpinellifolium* pairs of miR482/2118 members showed the same expression pattern at 2.2 ± 1.2 time-points (*p-*value = 0.012; figure 2*b*) and in *S. arcanum* at 2.2 ± 0.9 time-points (*p-*value = 0.005; figure 2*c*)*.* Lower co-regulation suggests additional gene-specific regulatory mechanisms in the wild tomatoes. By contrast, the cultivated tomato appears to have a more global co-regulation of miR482/2118 expression. These differences in co-regulation between wild and cultivated tomatoes could result from (i) differences in the evolutionary history of these plants (i.e. artificial versus natural selection) that brought about a more streamlined regulation of expression of miR482/2118 in *S. lycopersicum* or (ii) greater sensitivity to pathogen manipulation of host RNA silencing in *S. lycopersicum*, for example, due to pathogen-secreted RNA silencing suppressors. The latter is of interest because two RNA silencing suppressors have been previously described in *P. infestans* [26,50]. Additionally, we observed a substantial downregulation of additional miRNAs in *S. lycopersicum* that do not target *NBS-LRRs* (*Sl*miR156a/b/c, *Sl*miR166a/b, *Sl*miR168a/b and *Sl*miR172a/b) in *S. lycopersicum* from 24 hpi onwards (electronic supplementary material, figure S4)*.* Of these four, only *Sl*miR172a/b is implicated to function in *P. infestans* resistance, albeit by a different mechanism [51].

Next, we examined the relationship between the expression of miR482/2118 miRNAs and the life cycle of *P. infestans*. We focused on 6, 48 and 72 hpi, because they are critical time-points during infection by *P. infestans* and they correspond to the time frame when the greatest co-regulation between pairs of *Sl*miR482/2118 and their targets is detected (figure 3; electronic supplementary material, S2). We observed that six of the seven miRNAs were upregulated at 6 hpi (figures 2*a* and 3), which should result in enhanced suppression of their *NBS-LRR* targets. This was indeed true for three of the *NBS-LRR* targets screened: *Solyc02g036270.2.1, Solyc08g075630.2.1* and *Solyc08g076000.2.1*. The gene *Solyc02g036270.2.1* served as a reference *NBS-LRR,* because it was so far not reported to be associated with resistance to *P. infestans*.

We compared the expression patterns of *Sl*miR482/2118 with those in the close relatives of *S. lycopersicum*. In *S. pimpinellifolium*, only two *Sp*miR482/2118 members were significantly upregulated at 6 hpi (figure 2*b*). In *S. arcanum,* none of the seven *Sa*miR482/2118 members were upregulated at this time-point (figure 2*c*). Moreover, four out of seven *Sa*miR482/2118 were significantly downregulated at 6 hpi in *S. arcanum* (figure 2*c*), which was the most resistant tomato species. All of these members of miR482/2118 have targets associated with *P. infestans* defence in *S. lycopersicum* (electronic supplementary material, table S2). In fact, the *R*-gene targets of *Sl*miR482a and *Sl*miR482f were significantly downregulated at 6 hpi in *S. lycopersicum* (figure 3). Therefore, the downregulation of *Sa*miR482a and *Sa*miR482f upon infection in *S. arcanum* might be related to the enhanced resistance observed in this species. This downregulation of *Sa*miR482/2118 in *S. arcanum* in the presence of the pathogen could allow for an earlier response to the pathogen, because the predicted *NBS-LRR* targets would not be repressed during the first 6 h, as they are in *S. lycopersicum.* Taken together, these results point to miR482a and miR482f as potential regulators of *P. infestans-*associated defence responses.

Given the substantial co-regulation of miRNAs and their targets at 48 and 72 hpi, we evaluated the association of miR482/2118 expression with the life cycle progression of *P. infestans* on its hosts. In the biotrophic phase (prior to 72 hpi), *P. infestans* requires a living host. High R*-*protein activity during this time frame could lead to earlier pathogen perception and activation of HR/cell death, which in turn would limit pathogen spread [20]. In the necrotrophic phase, *P. infestans* induces host cell death [52–53]. High R*-*protein activity at this time-point may not be beneficial to the host, but instead benefit the pathogen. An effective plant resistance response during the necrotrophic phase may include the suppression of cell death-inducing proteins, such as R-proteins, perhaps through an upregulation of miR482/2118. By contrast, if pathogen-mediated RNA silencing suppression were effective at these later time-points, one would expect a downregulation of miRNAs, including miR482/2118.

At 48 hpi*,* four *Sl*miR482/2118 (*Sl*miR482f, *Sl*miR482, *Sl*miR482/h and *Sl*miR5300) were downregulated specifically in infected plants (figure 2*a*). While this does not exclude a plant-mediated downregulation of miR482/2118, the downregulation of the non-*NBS-LRR* regulating miRNAs (electronic supplementary material, figure S4), indicates that pathogen-mediated RNA silencing suppression may play a role here. In agreement with this, a *P. infestans* RNA silencing suppressor, potentially involved in silencing the miRNA-mediated silencing pathway, has its highest expression in the main biotrophic phase [26]. In *S. arcanum,* three *Sa*miR482/2118 members (*Sa*miR482a, *Sa*miR482b and *Sa*miR482h) were downregulated during the infection compared to the control (figure 2*c*). By contrast, none were downregulated in *S. pimpinellifolium* (figure 2*b*).

After the transition to necrotrophy at 72 hpi, the following miRNAs were upregulated: *Sl*miR482a, *Sl*miR482b and *Sl*miR482f in *S. lycopersicum*, *Sa*miR482a, *Sa*miR482h and *Sa*miR5300 in *S. arcanum* and *Sp*miR482a in *S. pimpinellifolium* (figure 2). None of the control *Sl*miRNAs were significantly upregulated (electronic supplementary material, figure S4), suggesting a miRNA-specific plant response at this time-point. miR482a is upregulated at 72 hpi in the infections across all three species, despite the small lag in *S. arcanum* for the transition from biotrophy to necrotrophy. This would suggest that upregulation of miR482a is a consistent phenotype associated with a plant defence response during the necrotrophic phase of *P. infestans*. This is further supported by the negative co-regulation of *Sl*miR482a and its target *Solyc11g06530.1.1* at this time-point (figure 3; electronic supplementary material, S2).

## 4. Conclusion

In this study, we investigated the expression of miR482/2118 during the infection of *P. infestans* on three different tomato species. We found that co-regulation of mature *Sl*miR482/2118 and their targets in cultivated tomato was highest during the initial phase of infection and during the life cycle transition of *P. infestans* from biotrophy to necrotrophy. Across-species comparisons of the gene expression of mature miR482/2118 and of the strength of resistance led to two main conclusions: (i) Co-evolution of *P. infestans* and *S. lycopersicum* may have resulted in a more efficient pathogen-mediated RNA silencing suppression compared with its more resistant sister species; and (ii) miR482a and miR482f could be identified as candidate miRNAs for mediating the resistance response of tomatoes to *P. infestans*.

## Supplementary Material

Supplementary Figures

## Supplementary Material

Table S1

## Supplementary Material

Table S2

## Data accessibility

The precursor sequences of the *Sa*miR482/2118 family, have been identified in the draft genome from *S. arcanum* based on a BLASTn approach. The sequence data are made available in the electronic supplementary material.
