# Supplementary material for: Expression profiling across wild and cultivated tomatoes supports the relevance of early miR482/2118 suppression for Phytophthora resistance
Source: Proc Biol Sci. 2018 Feb 28;285(1873):20172560. doi: 10.1098/rspb.2017.2560 (PMC5832704; doi:10.1098/rspb.2017.2560)
Supplement: Supplementary Figures [file rspb20172560supp1.pdf]

(*a*)

>*Sa*miR482a
AATTGGGAAAGGGATGAAGATTTTTGGGAAGCGTTTGGCGTGAGCGGTTGGGAAAGATAGATCTATCATGTTAGTATGATATGATAAATCTTGCCTACACCGCCCATGCCCATGGCTTC
CAATTATTTCTCCCTTTCCATACAATT

>*Sa*miR482b
GAAATAGAAAGGGGATGAGTGTTTCGAGGAGGTGTTGGAGTGGGTGGTGTGGTAAGATTTCATATTTATTTTCTATAAAAATTGAGATCTTGCCGATACCGCCCATTCCTATGGCTTCT
GATCATTTCTCTCTTTCCTTTTTC

>*Sa*miR482f
GGAAGTTGTTGGTTGTGGGTGGGGTGGAAAGATTTGATAAATCTAATTTTTTTTTAATTGAAAAAAAAAAATGGAAAAGAGATTAAATCTTTCCTACTCCTCCCATACCCATGACTTCC

>*Sa*miR482g
GAGGGATTTGGGAATCTTTTGGAGTGGGTGGGATGGAAAAATATTGTATTTATTTTTTTTCTTAAGGAAAAAAGATGAGAATTGAGATTTTTCCTATTCCACCCATGCCAATGGCTTCCA
GTCATTTACTC

>*Sa*miR482
GGAGATGAGTTTTTCGAAAATCTATGGGAATTGGTGAGTTGGAAAGCTTTTCTTTTTCTTCTCCTTCTCTTGTGATTAGCTTTCCAATTCCACCCATTCCTATGGTTTTTCGGTATTCTCTC
C

>*Sa*miR482h
GAAATGGGAGTTGAGCCCAACAAAAACTTTTGGGAATGGGTGGGTTGGTAAGCTTTTCCTTTTTTTATATGTGTGTTCCCTTAGCTTACCAATTCCACCCATTCCCATGGGTTTTTGGGC
TTTTCTCCTTTTTC

>*Sa*miR5300
TGACCAAAATTCTTCTTCCCCAGTCCAGGCATTCCAACAGTTGATAACATCTAGATCTGCTAATCCTTTGTATTTTTTGTTCTTCTATTTTTCACATGACATCTTCAAAATCCACTATGAGC
TTCATCATAAGCTAAGAAATGGTGAGTTTTGATGATGTGATGCCTAAAATAGGATTAAATGAAAGTACTAGTAAAAAGGTGTTCAAATCTGGAGGTTATGTTAAGCTGGTGGTATGCTTT
GATTGGGAAAGATGACTTAGGTCA

(*b*)

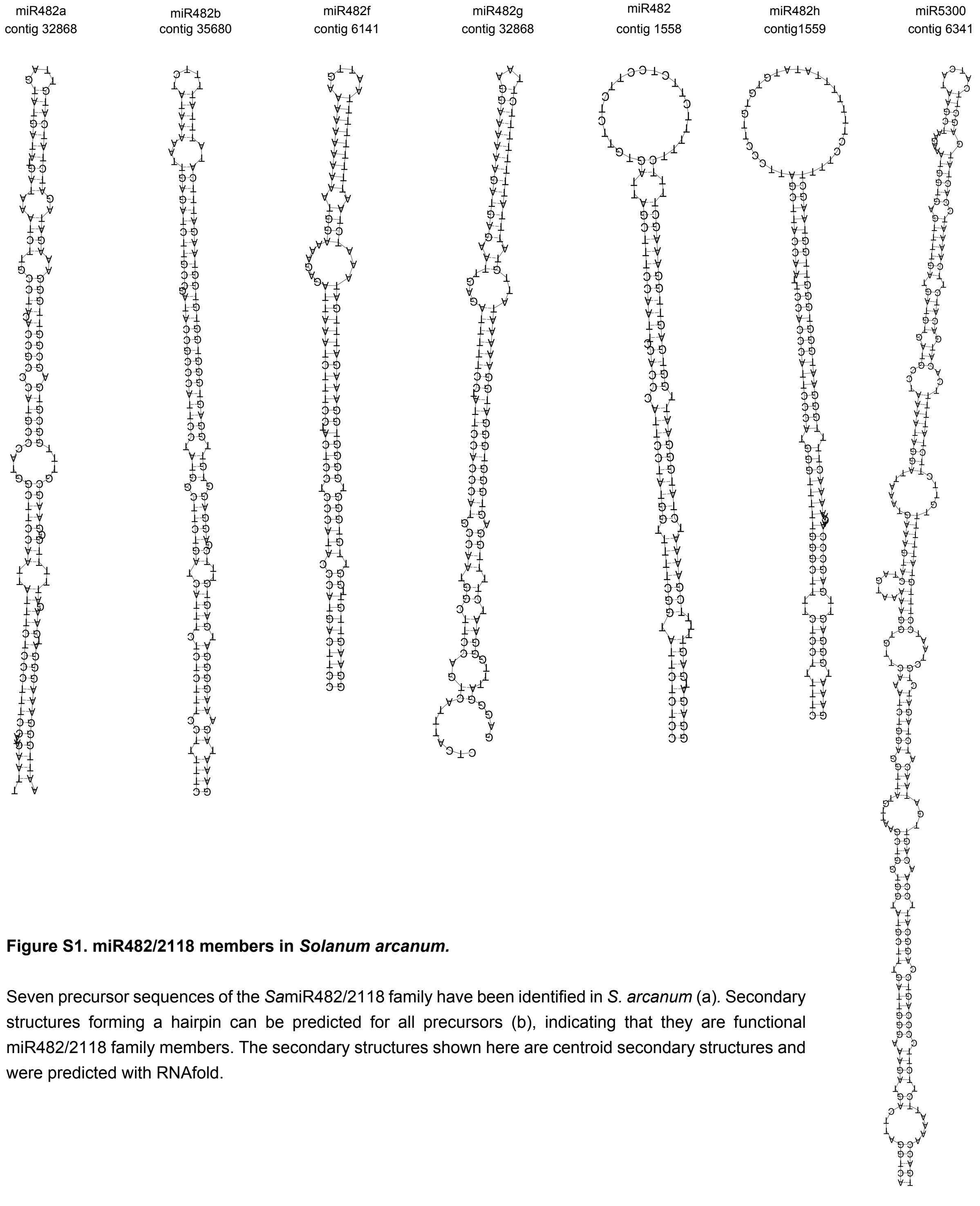

Figure S1. miR482/2118 members in *Solanum arcanum*.

Seven precursor sequences of the *Sa*miR482/2118 family have been identified in *S. arcanum* (a). Secondary structures forming a hairpin can be predicted for all precursors (b), indicating that they are functional miR482/2118 family members. The secondary structures shown here are centroid secondary structures and were predicted with RNAfold.

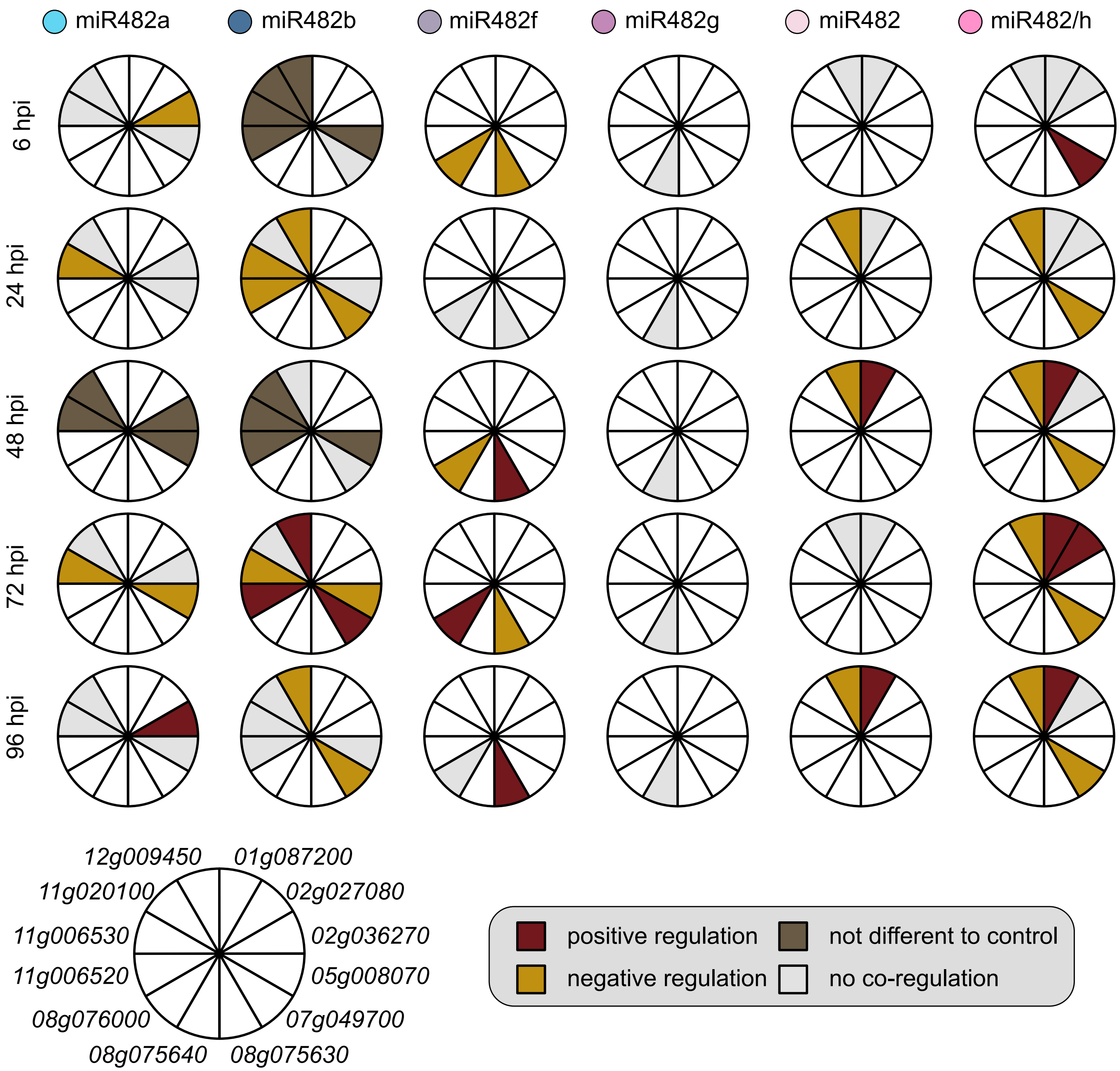

Figure S2. Co-regulation of *Sl*miR482/2118 and their targets. *NBS-LRRs* are represented in a circle as indicated by the circle in the bottom left. Three types of co-regulation are detected: negative co-regulation (yellow), positive co-regulation (red) or no differential regulation in infection vs. mock for both miRNA and target (brown). A lack of co-regulation is indicated in grey. Cells are left white if the miRNA is not predicted to target the *NBS-LRR.*

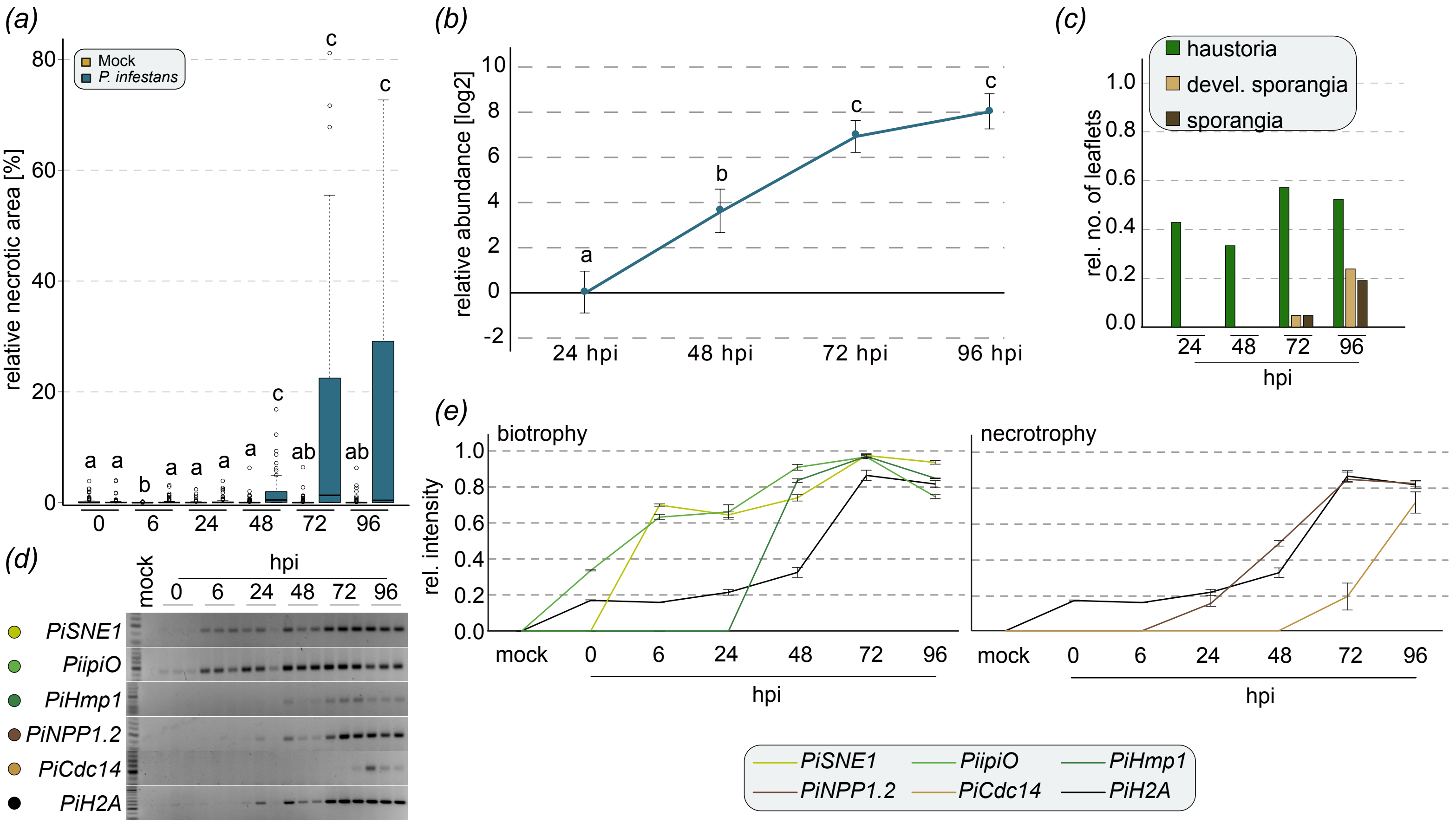

Figure S3. Infection progress of *P. infestans* in *S. arcanum*. (a) Relative necrotic area of leaflets of *S. arcanum* in mock controls (yellow) and inoculations with *P. infestans* (blue). The boxes represent 50 ± 25% of the data (interquartile range), the black lines in the boxes represent the median. The whiskers indicate 1.5x the interquartile range and outliers are marked as circles. (b) Relative abundance of *P. infestans* in *S. arcanum* 24 to 96 hpi (log2) was measured with a qRT-PCR of *PiH2a* with respect to three plant reference genes. The dots represent the average relative abundance and the error bars indicate the SEM. (c) Presence of haustoria (green), developing sporangia (beige) and mature sporangia (brown) of *P. infestans* 24 to 96hpi in *S. arcanum*. (d and e) Expression of markers genes for the biotrophic phase (green), the necrotrophic phase (brown) and the biomass (black) of *P. infestans* from mock and 0 to 96hpi in *S. arcanum* infections using RT-PCR. Error bars indicate the SEM. Statistical differences in all sub-figures are indicated with different letters. The p-value cut-off was 0.05.

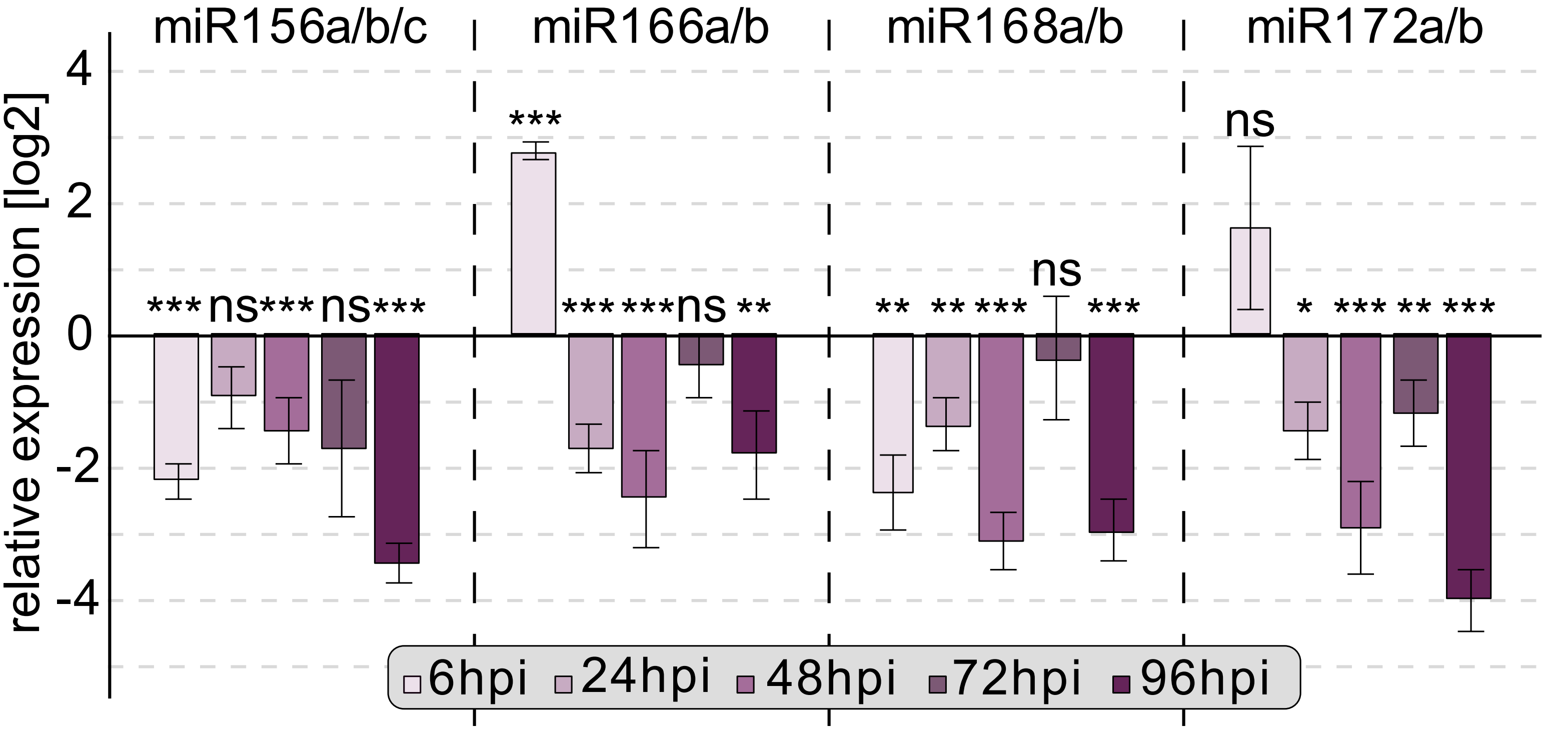

Figure S4. Expression of mature miRNAs in *Solanum lycopersicum*.
Relative expression (log2) of the mature miRNAs *Sl*miR156a/b/c, *Sl*miR166a/b, *Sl*miR168a/b and *Sl*miR172a/b in infections vs. mock from 6 to 96 hpi. The bars represent the average relative expression of the mature miRNAs and the error bars indicate the SEM. Significant differences of the relative expression of the miRNA in infection compared to mock at a specific time-point are indicated by * (p-value < 0.05), ** (p-value < 0.01), *** (p-value < 0.001), and ns (not significant).
