# Supplementary material for: Expression profiling across wild and cultivated tomatoes supports the relevance of early miR482/2118 suppression for Phytophthora resistance
Source: Proc Biol Sci. 2018 Feb 28;285(1873):20172560. doi: 10.1098/rspb.2017.2560 (PMC5832704; doi:10.1098/rspb.2017.2560)
Supplement: Table S1 [file rspb20172560supp2.docx]

**Table S1.** Primer sequences (5’ to 3’), PCR product length, gene specific annealing temperature and gene specific elongation time for primers used in the 5’ RLM-RACE and qRT-PCR. [14] – Primers are from Ouyang et al. 2014

| **Name** | **Sequence** | **Length [bp]** | **Annealing Temperature [˚C]** | **Elongation time [s]** | **Usage** |
| --- | --- | --- | --- | --- | --- |
| Solyc02g036270.2.1 | >GeneRacerTM 5' primer  - supplied in kit -  >Gene specific primer  5’ ACATCTCGGAGTCGCGTTGT 3’ | 374 | 60 | 25 | Nested 5’RLM-RACE; step 1 |
| Solyc08g075630.2.1  **[14]** | >GeneRacerTM 5' primer  - supplied in kit -  >Gene specific primer  5’ CCAAATGCACGTTGCATGAACA 3’ | 467 | 60 | 30 | Nested 5’RLM-RACE; step 1 |
| Solyc08g076000.2.1 | >GeneRacerTM 5' primer  - supplied in kit -  >Gene specific primer  5’ TGGCCGCTAAAGGCACACCACCACAT 3’ | 550 | 60 | 35 | Nested 5’RLM-RACE; step 1 |
| Solyc02g036270.2.1 | >GeneRacerTM 5' Nested primer  - supplied in kit -  >Gene specific nested primer  5’ CTGATCTCCACGATTCCAGA 3’ | 211 | 63 | 15 | Nested 5’RLM-RACE; step 2 |
| Solyc08g075630.2.1  **[14]** | >GeneRacerTM 5' Nested primer  - supplied in kit -  >Gene specific nested primer  5’ GGGTAGTGGTTAGAACATAAGC 3’ | 350 | 62 |  | Nested 5’RLM-RACE; step 2 |
| Solyc08g076000.2.1 | >GeneRacerTM 5' Nested primer  - supplied in kit -  >Gene specific nested primer  5’ GCGACGTTCCCATAATTGATCCA 3’ | 386 | 62 | 25 | Nested 5’RLM-RACE; step 2 |
| Solyc01g087200.2.1 | >Forward  5’ CCCGAGTTAGGTCTCGCTTC 3’  >Reverse  5’ ACATCTTGTCACATCGTCCCA 3’ | 239 | 63 | Na | qRT-PCR |
| Solyc02g036270.2.1 | >Forward  5’ CCACGATAGTTCGCTCATGGA 3’  >Reverse  5’ GCAACTCGGTCAGCACATTC 3’ | 155 | 60 | Na | qRT-PCR |
| Solyc02g027080.1.1 | >Forward  5’ TGTCAGCAACAAAAGTCGAGA 3’  >Reverse  5’ TCACCAGTCCAGCGCTTAAA 3’ | 240 | 62 | Na | qRT-PCR |
| Solyc05g008070.2.1 | >Forward  5’ ACTCTTGCGAGCTAAATCACC 3’  >Reverse  5’ AGCCTTTTCGCGATCGTCTT 3’ | 250 | 63 | Na | qRT-PCR |
| Solyc07g049700.1.1 | >Forward  5’ GACGGACACCCGATAATTGGA 3’  >Reverse  5’ TGCCACCACTTCTAACCTTGT 3’ | 248 | 65 | Na | qRT-PCR |
| Solyc08g075630.2.1 | >Forward  5’ AAAGAGCTGCCAAACAGCCT 3’  >Reverse  5’ GCCAGTGTTGGACATTCCCTA 3’ | 212 | 60 | Na | qRT-PCR |
| Solyc08g075640.2.1 | >Forward  5’ TGCGTAGTCTTCCAGAGCAG 3’  >Reverse  5’ CCTCCTCCCCACTGCAAATC 3’ | 218 | 65 | Na | qRT-PCR |
| Solyc08g076000.2.1 | >Forward  5’ AAGTCTACGGAAGGGACAAAGAG 3’  >Reverse  5’ TTTGGGATCGAAATGCTTAGTC 3’ | 176 | 63 | Na | qRT-PCR |
| Solyc11g006520.1.1 | >Forward  5’ TTCTTCGCCAGCTATGCCTAA 3’  >Reverse  5’ TCATTGCTATGCGCTGGACG 3’ | 155 | 66 | Na | qRT-PCR |
| Solyc11g006530.1.1 | >Forward  5’ GGTTAGAAGTGGTTGCGGAG 3’  >Reverse  5’ TGCGAGTTATACGACTGCTCTG 3’ | 228 | 64 | Na | qRT-PCR |
| Solyc11g020100.1.1 | >Forward  5’ ACCTAACCAATCTTTCGTTTGTC 3’  >Reverse  5’ TTCCTCAAACAACTTACAAGCCC 3’ | 247 | 65 | Na | qRT-PCR |
| Solyc12g009450.1.1 | >Forward  5’ GATTGCGGAGGGGTTCTTGA 3’  >Reverse  5’ TCGATATCCCACTTGAGGAACG 3’ | 241 | 67 | Na | qRT-PCR |
| *Sl*miR482a/ *Sp*miR482a/ *Sa*miR482a | >Forward  5’ TCTTGCCTACACCGCCCATGCC 3’  >Reverse  - supplied in kit - | Na | 65 | Na | qRT-PCR |
| *Sl*miR482b/ *Sp*miR482b | >Forward  5’ TCTTGCCAATACCGCCCATTCC 3’  >Reverse  - supplied in kit - | Na | 66 | Na | qRT-PCR |
| *Sa*miR482b | >Forward  5’ TCTTGCCGATACCGCCCATTCC 3’  >Reverse  - supplied in kit - | Na | 66 | Na | qRT-PCR |
| *Sl*miR482f/ *Sp*miR482f/ *Sa*miR482f | >Forward  5’ TCTTTCCTACTCCTCCCATACC 3’  >Reverse  - supplied in kit - | Na | 55 | Na | qRT-PCR |
| *Sl*miR482g/ *Sp*miR482g/ *Sa*miR482g | >Forward  5’ TTTCCTATTCCACCCATGCCAA 3’  >Reverse  - supplied in kit - | Na | 64.4 | Na | qRT-PCR |
| *Sl*miR482/ *Sp*miR482/ *Sa*miR482 | >Forward  5’ TTTCCAATTCCACCCATTCCTA 3’  >Reverse  - supplied in kit - | Na | 55 | Na | qRT-PCR |
| *Sl*miR482h/ *Sp*miR482h | >Forward  5’ TTACCAATTCCACCCATTCCTA 3’  >Reverse  - supplied in kit - | Na | 55 | Na | qRT-PCR |
| *Sa*miR482h | >Forward  5’ TTACCAATTCCACCCATTCCCA 3’  >Reverse  - supplied in kit - | Na | 55.7 | Na | qRT-PCR |
| *Sl*miR5300/ *Sa*miR5300 | >Forward  5’ TCCCCAGTCCAGGCATTCCAAC 3’  >Reverse  - supplied in kit - | Na | 55 | Na | qRT-PCR |
| *Sp*miR5300 | >Forward  5’ TCTCCAGTCCAGGCATTCCAAC 3’  >Reverse  - supplied in kit - | Na | 55 | Na | qRT-PCR |
| *Sl*miR390a/ *Sp*miR390a/ *Sa*miR390a | >Forward  5’ AAGCTCAGGAGGGATAGCACC 3’  >Reverse  - supplied in kit - | Na | 60.9 | Na | qRT-PCR |
| *Sl*miR156a/b/c | >Forward  5’ TTGACAGAAGATAGAGAGCAC 3’  >Reverse  - supplied in kit - | Na | 59 | Na | qRT-PCR |
| *Sl*miR166a/b | >Forward  5’ TCGGACCAGGCTTCATTCCCC 3’  >Reverse  - supplied in kit - | Na | 59 | Na | qRT-PCR |
| *Sl*miR168a/b | >Forward  5’ CCCGCCTTGCATCAACTGAAT 3’  >Reverse  - supplied in kit - | Na | 57 | Na | qRT-PCR |
| *Sl*miR172a/b | >Forward  5’ AGAATCTTGATGATGCTGCAT 3’  >Reverse  - supplied in kit - | Na | 59 | Na | qRT-PCR |
